# Pilot-Scale Oxygen-Balanced Mixotrophic Cultivation of *Galdieria sulphuraria*

**DOI:** 10.1021/acssuschemeng.4c09186

**Published:** 2025-01-31

**Authors:** Pedro Moñino Fernández, Marina López Morales, Aniek de Winter, Fred van den End, Marcel Janssen, Maria Barbosa

**Affiliations:** Bioprocess Engineering, AlgaePARC, Wageningen University and Research, P.O. Box 16, 6700 AA Wageningen, The Netherlands

**Keywords:** mixotrophy, tubular photobioreactor, natural light, control, microalgae

## Abstract

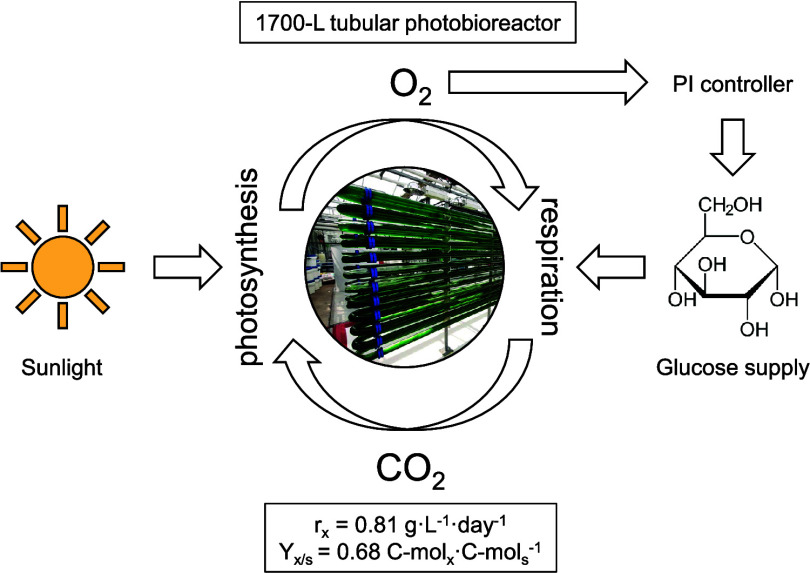

Oxygen-balanced mixotrophy (OBM) is a particular type of microalgae mixotrophic cultivation, where the supply of an organic carbon substrate is adjusted to match heterotrophic oxygen consumption with photosynthetic production. In this way, the need for aeration is eliminated due to intracellular gas recycling during daytime. After implementing this process at lab scale, we sought to explore its scalability in a tubular photobioreactor (TPBR). In this study, OBM was implemented in a two-phase tubular photobioreactor of 1700 L placed in a greenhouse and exposed to sunlight. The process was run with the polyextremophilic species *Galdieria sulphuraria*, using glucose as a carbon source. The gas phase was continuously recirculated, and the oxygen concentration was monitored and utilized to manage the glucose supply through a proportional-integral controller. An excessive rate of night aeration, however, resulted in CO_2_ limitation issues. Subsequent tuning and optimization of controller settings and the nighttime aeration rate effectively addressed the problem. The average biomass productivity reached 0.81 g·L^–1^·day^–1^, a significant improvement over autotrophic productivity in the same pilot system. On the other hand, the biomass yield on the substrate was 0.68 C-mol_*x*_·C-mol_s_^–1^, indicating that considerable carbon recycling took place but to a lower extent than at lab scale. These results provide a solid foundation for the large-scale industrial implementation of OBM.

## Abstract 2

This work explores the implementation of oxygen-balanced mixotrophy at an industrially relevant scale, enhancing the sustainability and economics of microalgae cultivation by reducing aeration needs and improving biomass productivity.

## 1 Introduction

Oxygen-balanced mixotrophy (OBM) is a novel type of microalgae cultivation that combines the advantages of mixotrophic growth with precise control over oxygen (O_2_) and carbon dioxide (CO_2_) dynamics.^1^ Mixotrophic metabolism per se is the integration of the photoautotrophic and chemoheterotrophic machineries within a single organism. Thus, both light and an organic carbon substrate are utilized as energy source and both inorganic and organic carbon as carbon source.^2^ The term “oxygen-balanced” refers to a state where the oxygen consumed equals the oxygen produced.^3^ Given a specific light supply, precise control over the oxygen balance is achievable by adjusting the supply of an organic carbon substrate. When the appropriate amount is supplied, photosynthetic oxygen production is similar to that of the oxygen consumed through intracellular recycling.

This precise balancing extends to CO_2_ as well, as the CO_2_ released during the oxidation of the organic carbon substrate is reintroduced in the metabolic network by the Calvin–Benson–Bassham cycle. The assimilation of CO_2_ results in the production of O_2_ as a byproduct of photosynthesis, which can again fuel oxidation of the organic substrate. This closed-loop mechanism eliminates the need for external gas input, allowing the system to operate seamlessly during daytime.^4^ While perfect CO_2_ balancing cannot be achieved due stoichiometric differences between O_2_ and CO_2_, carbon recycling efficiency reaches up to 90%.^1^ Moreover, the addition of an organic carbon source reduces dependence on light availability, which is a significant limitation in mass autotrophic cultivation of microalgae. This feature enables OBM to achieve higher volumetric biomass productivities compared to autotrophic metabolism.

The implementation of this bioprocess in the laboratory was started by monitoring dissolved oxygen (DO) levels in the medium. The DO signal was linked to a controller that adjusts the supply of the organic substrate into the reactor, a stirred-tank vessel. Through this approach, DO could be consistently maintained at a stable level. However, scaling up this process to larger reactors, particularly those designed for phototrophic growth, such as tubular photobioreactors (TPBRs), poses challenges. The increase in volume and the tubular geometry in these large-scale systems introduce a higher potential for fluctuations of both oxygen and organic substrate levels.^5^

In order to derisk the scale-up, we conducted a prior study to investigate these fluctuations in a lab setting that simulated a TPBR.^6^ The study identified the depletion of DO as a critical factor, affecting pigment synthesis and the overall process performance. These findings indicated that to mitigate such issues in an actual scale-up, efforts should focus on minimizing fluctuations in DO.

In this context, designing an adequate automatic glucose supply for TPBR is challenging. From the operator’s perspective, understanding and interpreting the response of such a complex system is hindered by the intricate interplay of biological, chemical, and physical phenomena. Computational tools can support control design, particularly by using models of the TPBR to evaluate and fine-tune glucose supply control strategies.^7^ Although these tools serve as a valuable starting point, in vivo testing and optimization remain essential.

Previously, we have implemented OBM with the red microalga *Galdieria sulphuraria*.^8^ This species is able to utilize various organic carbon substrates, either heterotrophically or mixotrophically.^9,10^ This ability provides considerable versatility in substrate selection, an advantageous trait for the potential scale-up with waste streams. In addition, the polyextremophilic nature of *G. sulphuraria* allows for the use of cultivation conditions that are unsuitable for many other microorganisms, reducing the risk of contamination.^11^ Our previous findings indicate that this species contains a high protein content when growing mixotrophically, including the blue phycobiliprotein C-phycocyanin, a high-value pigment.^12^ Such composition suggests commercial potential for the biomass of *G. sulphuraria* produced via OBM, making this species valuable beyond the technical aspects of the cultivation. Taking these attributes into account, *G. sulphuraria* is considered an ideal candidate for scaling up OBM.

Building upon our earlier research, in this work we scaled up OBM to a pilot two-phase TPBR using *G. sulphuraria*. In two-phase systems, mixing is achieved through gas injection, resulting in a low energy footprint. Furthermore, mass transfer occurs across the tubular section, as the gas and liquid phases move concurrently. In this context, the gas phase can serve as an oxygen reservoir, providing a supplemental oxygen supply during periods of high demand. Our approach involved using the concentration of oxygen in the gas phase to adjust organic carbon supply as it showed smoother oscillations than the DO. Therefore, the control system is less susceptible to amplify noise in the signal. Glucose was selected as a standard carbon substrate to illustrate the capabilities of the OBM. Although it may not be the most cost-effective substrate for large-scale implementation, it provides a simple and practical starting point. This allows us to demonstrate the principle of OBM, which is the primary objective of this work. Glucose supply was regulated by a proportional-integral-intermediate (PI) controller optimized throughout the experiment. The effectiveness of the PI controller was monitored by measuring the concentration of glucose, the oscillatory range of DO and a marker of anoxia, coproporphyrin III (COPROIII). Additionally, the performance of the process, measured as the biomass productivity and the biomass yield on substrate, was analyzed. The results were compared with the results obtained previously on the lab scale to assess the feasibility of OBM for large-scale production. Biomass composition analyses were not included in this study, as the main focus was process development.

## 2 Materials and Methods

### 2.1 Strain and Medium

*G. sulphuraria* ACUF 064 (http://www.acuf.net) was kindly provided by Prof. A. Pollio (University of Naples, Italy). The medium used for the cultivation of this strain consisted of the following components (expressed in mol·L^–1^): 8.0 × 10^–2^ (NH_4_)_2_SO_4_, 6.5 × 10^–3^ MgSO_4_·7H_2_O, 4.7 × 10^–4^ CaCl_2_·2H_2_O, 6.3 × 10^–4^ FeCl_3_·6H_2_O, 2.0 × 10^–4^ Na_2_EDTA·2H_2_O, 1.2 × 10^–2^ H_3_PO_4_, 1.7 × 10^–3^ NaCl, 8.1 × 10^–3^ KCl, 8.0 × 10^–4^ H_3_BO_3_, 8.1 × 10^–5^ MnCl_2_·4H_2_O, 8.2 × 10^–5^ ZnCl_2_, 3.2 × 10^–5^ CuSO_4_·5H_2_O, 1.7 × 10^–5^ Na_2_MoO_4_·2H_2_O, and 1.7 × 10^–5^ CoCl_2_·6H_2_O. pH was adjusted to 1.8 with H_2_SO_4_. This composition was designed to ensure that all components were in excess, preventing any limitations in inorganic nutrients.

### 2.2 Inoculum Chain

Cryopreserved stock cultures were cultivated in four 250 mL flasks, each containing 100 mL of medium, at 37 °C, 2% v/v CO_2_, and 120 rpm. The photon flux density (PFD) was set at 100 μmol·m^–2^·s^–1^, following a day/night cycle block pattern of 16:8 h. These flasks served as the inoculum for a 25 L two-phase tubular photobioreactor (TPBR), the Lgem Lab-25 (Lgem, The Netherlands), with an initial biomass concentration (*C*_*x*_) of 0.18 g·L^–1^ on the 30th of May 2023. The system was operated with a working volume of 18 L and a continuous aeration rate of 6 L·min^–1^ enriched with 10% CO_2_. The reactor was illuminated with artificial light following a day/night cycle sinusoidal pattern of 16:8 h. The peak light intensity was progressively increased to 500 μmol·m^–2^·s^–1^ to promote faster growth and to adapt the cells to higher PFDs. The temperature was maintained within 30–40 °C. After a week, the culture had reached a *C*_*x*_ of 3.5 g·L^–1^ and was transferred to a 300 L horizontal single-phase TPBR with a separate degasser. This system, operated with a working liquid volume of 250 L, utilized natural light, and the degasser was aerated at 10 L·min^–1^ enriched with 5% v/v CO_2_. Temperature was maintained within 25–40 °C. The cultivation continued for 20 days, concluding on the 26th of June 2023. This culture was used to inoculate the pilot-scale photobioreactor.

### 2.3 Photobioreactor Setup

**Figure 1:**
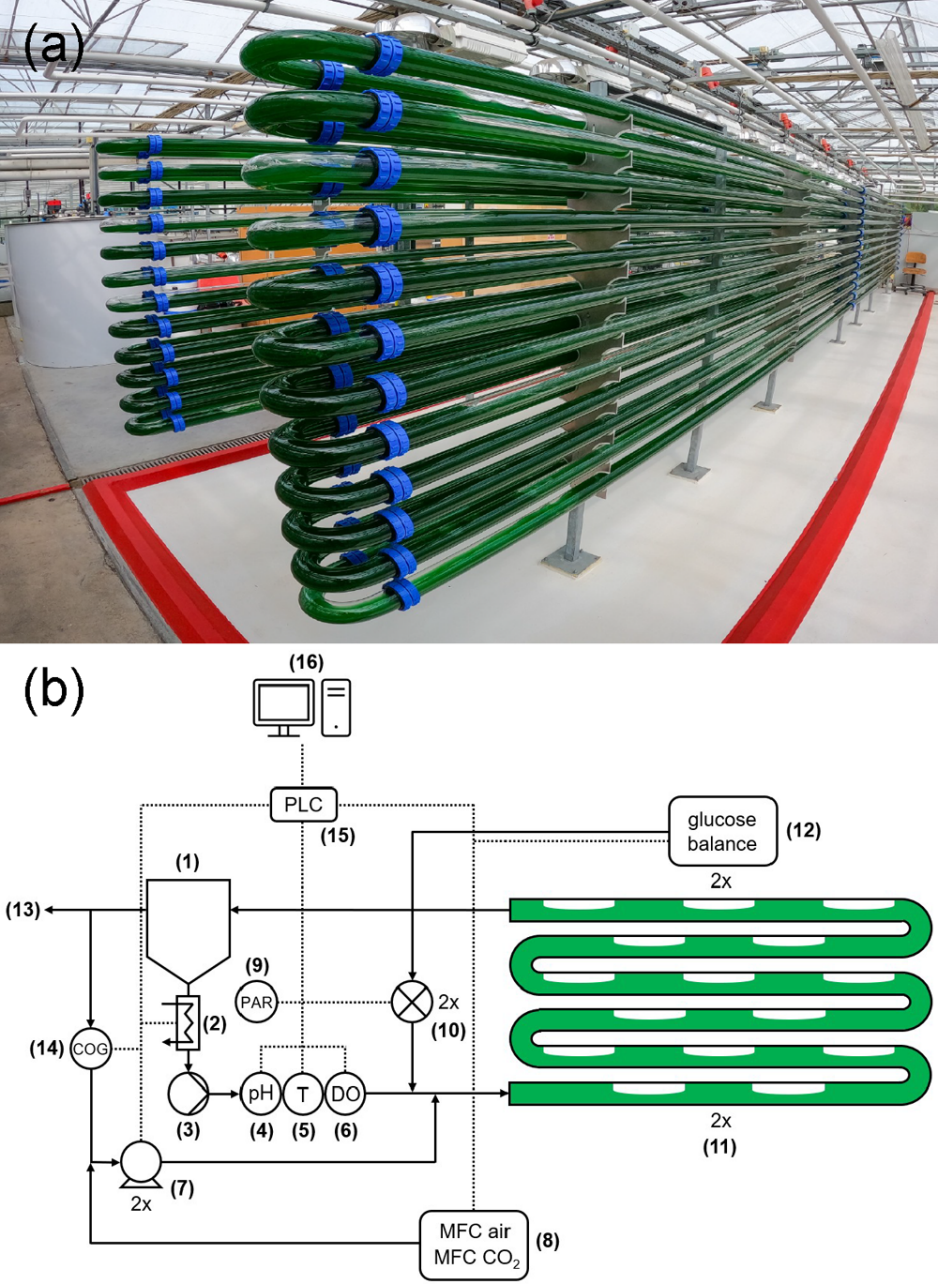
Oxygen-balanced mixotrophic cultivation of *G. sulphuraria* at pilot scale. (a) Two-phase tubular photobioreactor of 1700 L. (b) Schematic representation of reactor configuration, control, and data acquisition. The numbers indicate different parts of the system: (1) collection vessel, (2) heat exchanger, (3) liquid recirculation pump, (4) pH probe, (5) thermometer, (6) dissolved oxygen probe, (7) membrane gas pumps, (8) mass flow controller of air and CO_2_, (9) PAR sensor, (10) glucose pumps, (11) tubular section, (12) glucose bottle balance, (13) bleed gas, (14) oxygen in the gas probe, (15) programmable logic controller (PLC), and (16) data acquisition computer.

The experiment was carried out in a two-phase TPBR, the GemTube MK-1 1500s (Lgem, The Netherlands), located within a greenhouse with partial temperature control at our pilot facility AlgaePARC (Bennekom, The Netherlands). A picture of the TPBR and a scheme of the configuration are displayed in Figure 1. In total, the TPBR has an approximate volume of 1700 L and was operated with a working liquid volume of 1280–1350 L and a gas holdup of 0.20–0.25. The tubular section consists of two symmetrical helicoidal glass tubes of length 280 m and internal diameter 6.2 cm. Both helices are connected to a collection vessel, directing recirculated liquid to the bottom of the tubes by means of a liquid recirculation pump. Following the collection vessel are a heat exchanger with a heating capacity of 9 kW and sensor ports with probes for pH, temperature, and DO. The heat exchanger also provided cooling when it was required. The DO was measured using an optical probe, model VisiFerm DO ECS 120 (Hamilton). The gas phase is directed from the vessel toward two membrane gas pumps and then injected back into the tubes at the lowest part of the helices. Because of this, an elongated bubble regime is created that facilitates mass transfer along the tubular section. During the experiment, the recirculated gas flow rate in each helix was maintained within the range of 20–30 L·min^–1^. Under these conditions, liquid velocity was on the order of 0.4 m·s^–1^.

A PAR quantum sensor was positioned between the start of the left and right helices to measure the incoming PFD. The probe that measures the oxygen concentration in the gas phase (*C*_OG_) is positioned before the gas pumps. This probe was an equivalent model to the probe used for measuring DO. The *C*_OG_ measurement was calibrated based on air saturated with water vapor. The excess off-gas leaves the vessel via an exit equipped with a filter. Glucose is pumped into both helices at injection points located between the sensor ports and entry points to the two tubular helices. The concentrated glucose solution is placed on a balance allowing continuous monitoring of the glucose supply rate to each helix. Two mass flow controllers regulate air input and CO_2_ before the gas compressors, when required for autotrophic operation. A programmable logic controller (PLC) unit S7 1214 C (Siemens, Germany) connects and controls the probes, pumps, and other components of the system. Data visualization and logging are managed through a LabView virtual instrument (National Instruments) running on a computer connected to the PLC.

### 2.4 Pilot-Scale Oxygen-Balanced Mixotrophic Cultivation of *G. sulphuraria*

The 1700 L TPBR was inoculated on the 26th of June 2023. Initially, the reactor was covered with a translucent green cloth for 1 day to prevent photoinhibition. During the first week after inoculation, the TPBR was operated autotrophically to allow the cells to adapt to the new flow conditions. During this period there was an input airflow rate, and consequently the equivalent bleed flow rate, at 10 L·min^–1^, both during the day and night. During daylight hours, the air was enriched with 10% v/v of CO_2_. On the third of July, the transition to oxygen-balanced mixotrophy (OBM) began by pumping glucose solution of 200 g·L^–1^ at a fixed rate of 1.1 mL·min^–1^. This solution was not sterilized, as the modifications made to the TPBR for mixotrophic growth could not ensure sterility. Instead, it was changed daily, and the containers were cleaned with ethanol after each change to prevent the growth of contaminants. During this period, air input conditions remained consistent with the autotrophic phase. On the 5th of July, the OBM started and daytime input aeration was switched off. Gas recirculation continued through the gas pumps, exclusively recycling the gas phase since there was no further air input into the system.

Due to net CO_2_ production resulting from OBM, there was a minor bleed flow rate during this period, although it was not quantified. The 200 g·L^–1^ glucose solution supply was controlled automatically based on the oxygen concentration in the gas (*C*_OG_) and was automatically stopped when light input went below 50 μmol·m^2^·s^–1^. Nighttime airflow input was initially maintained at 10 L·min^–1^ and was adjusted later based on the respiratory demand of the cells during the experiment. This phase lasted for 6 days until the 11th of July. At this point, the TPBR was diluted, and the second batch commenced. The TPBR was operated autotrophically for the remainder of the day to prevent glucose accumulation after a sudden change in the specific light supply. The next day, fixed glucose feeding was reinstated at 2 mL·min^–1^ with aeration. On the 13th of July, automatic glucose supply was activated, initiating the OBM phase of the second batch. This phase continued until the 21st of July, spanning 8 days. The reactor underwent dilution again, and after 1 day of autotrophic operation, automatic glucose control was activated and proceeded until the 28th of July.

### 2.5 Controller Settings

Glucose supply during the OBM phases was automated by using a proportional-integral (PI) controller. The control strategy involved continuous control action as adjustments in the manipulated variable (MV) were made every 5 s. The MV was the percentage of glucose pump output with similar settings for both the west and the east glucose pumps. It ranged from 0%, equivalent to a flow rate of glucose solution of 0 mL·min^–1^, to 100%, equivalent to 20 mL·min^–1^ per pump. The set point was not defined by the operator; it depended on *C*_OG_. The value of *C*_OG_ was continuously compared with its value at the previous retention time (τ, s), but only after the completion of an initial pulse with a duration of τ_p_ (s). The error (*e*) was then calculated accordingly
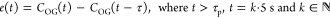
1The PI controller was implemented with initial values of 80 s for the proportional gain coefficient (*K*_P_) and 2000 s for the integral term (τ_I_). These choices were based on previous optimization in silico of the TPBR and its control.^7^ The initial pulse of 200 g·L^–1^ glucose solution was set for a τ_p_ of 10 min at 2 mL·min^–1^ per pump. The initial values for *K*_P_ and τ_I_, and the initial glucose pulse were adjusted during the experiment to further calibrate the response of the system. When the light received by the PAR sensor fell below 50 μmol·m^–2^·s^–1^, the controller was deactivated, and the supply of glucose was stopped. Once the light intensity surpassed 100 μmol·m^–2^·s^–1^ the controller was reactivated, initiating with a pulse.

### 2.6 Autotrophic Pilot-Scale Cultivation of *G. sulphuraria*

*G. sulphuraria* ACUF 064 was cultivated autotrophically in the GemTube MK-1 1500s (Lgem, The Netherlands) located at AlgaePARC (Bennekom, The Netherlands). The experiment took place during the summer of 2021 for a total of 87 days, spanning from the 25th of June to the 20th of September. The two-phase reactor, operated with a working liquid volume of 1280–1350 L, utilized both a single gas compressor and a liquid recirculation pump for gas and liquid recirculation, respectively. These settings resulted in a recirculation gas flow rate of 20 L·min^–1^ and a liquid velocity of nearly 0.4 m·s^–1^. Besides gas recirculation, an airflow input of air enriched with 10% v/v CO_2_ was active during daytime and nighttime at 10 L·min^–1^, accompanied by an equivalent gas bleed flow rate. The temperature was maintained between 25 and 45 °C by means of a heat exchanger with a heating capacity of 3 kW and greenhouse temperature partial control. The heat exchanger also provided cooling when required. No active pH control was employed. Regular offline measurements were performed for monitoring, and contamination was checked weekly. DO and *C*_OG_ were monitored by online measurements.

### 2.7 Dry Weight (DW) Concentration

*C*_*x*_ was determined through biomass dry weight (DW, g·L^–1^) analysis in technical triplicates. Fresh culture aliquots (1–5 mL) were diluted to 30 mL with demineralized water and filtered using preweighed Whatman GF/F glass microfiber filters (diameter of 55 mm, pore size of 0.7 μm). The filters were washed with 30 mL of deionized water and dried at 100 °C for a minimum of 3 h.

### 2.8 Average Absorption Cross Section

The average absorption cross section (*a*_*x*_, m^2^·kg_*x*_^–1^) in the PAR region (400–700 nm) of the spectrum was determined following the methodology determined in de Mooij et al.^13^ Briefly, fresh samples from the reactor were diluted to a concentration in the range of 0.5–2 g·L^–1^ DW and transferred to 2 cuvettes with an optical path of 2 mm. Absorbance measurements were conducted with a UV–vis/double-beam spectrophotometer (Shimadzu, Japan) equipped with an ISR-2600 integrating sphere ISR-2600.

### 2.9 Photosystem II Quantum Yield (QY)

Fresh biomass samples were diluted to an optical density (OD) at 750 nm ranging from 0.3 and 0.8. These samples were incubated in darkness at 35 °C for 20 min in triplicate. The dark-adapted photosystem II maximum quantum yield of photochemistry (QY, *F*_v_/*F*_m_) was measured at 455 nm with an AquaPen-C AP-C 100 instrument (Photon Systems Instruments, Czech Republic).

### 2.10 Coproporphyrin III Monitoring

Culture aliquots were sampled from the reactor at various time points across all batches and centrifuged at >20,000 RCF for 10 min. The resulting supernatant was immediately used for triplicate measurements of OD at 400 nm.

### 2.11 Total Carbon Determination

Culture aliquots were sampled during the OBM phase of the experiment and centrifuged for 10 min at >20,000 RCF. The pellets were washed twice with deionized water before being stored at −20 °C. Prior to measurement, the pellets were thawed at room temperature and analyzed for total carbon (TC, g·L^–1^) in triplicate with a TOC-L analyzer (Shimadzu, Japan). The biomass carbon content (*C*_%_, %w_C_·w_*x*_^–1^) was calculated by dividing TC by DW of the corresponding sample.

### 2.12 Glucose Quantification

Culture aliquots were sampled at various time points throughout the experiment and centrifuged for 10 min at >20,000 RCF. Glucose concentration was measured in the resulting supernatant using a YSI 2950 Biochemistry Analyzer (YSI Life Sciences).

### 2.13 Assessment of Contamination

Contamination assessment was conducted weekly during the experiment. Culture samples were stained with SYBR Green I (Sigma-Aldrich) and examined under fluorescence microscopy with an EVOS FL auto microscope (Thermo Fisher Scientific). The presence of fluorescent bacterial or fungal cells is easily detectable by the difference in size and shape compared to algal fluorescent cells.

### 2.14 Light Data

Incident shortwave radiation data for June and July 2023 were obtained from the De Veenkampen weather station in Wageningen (The Netherlands), located at 51° 58′ 52.309″ N 5° 37′ 13.109″ E. The data was retrieved as daily averages.

### 2.15 Biomass Productivity Calculation

The biomass volumetric productivity (*r*_*x*_, g·L^–1^·day^–1^) of each batch was determined through a linear regression analysis of the DW measurements. Only the measurements corresponding to the OBM periods were considered for analysis. The overall *r*_*x*_ value was derived by averaging the calculated productivity results across the three batches.

### 2.16 Mixotrophic Biomass Yield on Substrate Calculation

To begin, the total carbon-based amount of biomass produced during the OBM phases of the experiment was calculated (*M*_*x*_, C-g)
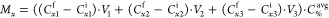
2In this equation, *C*_*x*_^f^ and *C*_*x*_^i^ represent the biomass concentrations at the end and beginning of the OBM phase for each batch, respectively (g·L^–1^). The subscripted number denotes the specific batch. *V* stands for the total liquid volume at the beginning of each batch (L). Volume changes resulting from evaporation and sampling were neglected for the sake of simplicity, as these changes did not exceed 6% in any batch. Lastly, *C*_%_^avg^ is the average biomass carbon concentration during the OBM phases of the experiment. Then, the total carbon-based amount of glucose consumed by the cells (*M*_s_, C-g) during the OBM phases of the experiment was determined
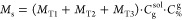
3where *M*_T_ (g) represents the total amount of 200 g·L^–1^ solution supplied to the reactor per batch, *C*_g_^sol^ stands for the glucose concentration in the glucose solution (g·L^–1^), and *C*_%_^g^ indicates the carbon fraction of glucose (% w/w). The mixotrophic biomass yield on the substrate (*Y*_*x*/s_^mixo^, C-mol_*x*_·C-mol_s_^–1^) was thereafter calculated
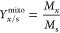
4

### 2.17 Statistical Procedures

The propagation of errors for addition operations was determined with eq 5
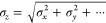
5

Similarly, the propagation of errors for multiplication operations was calculated with eq 6
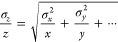
6Here, σ_*x*_ is the standard deviation associated with the value *x* and so on.

## 3 Results and Discussion

### 3.1 Pilot-Scale Mixotrophic Cultivation of *G. sulphuraria*

**Figure 2:**
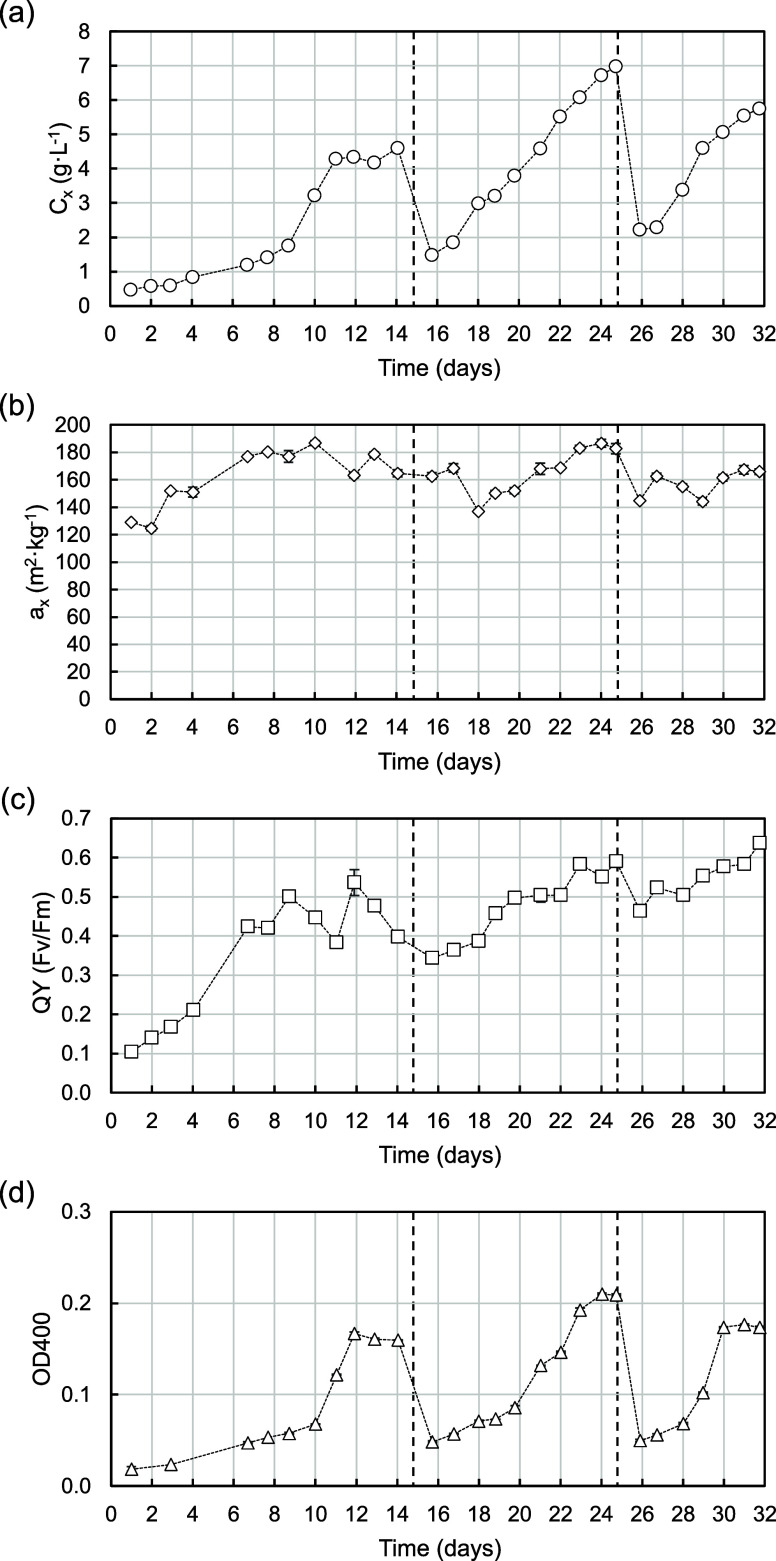
Offline cultivation parameters during pilot-scale mixotrophic cultivation of *G. sulphuraria* in the summer of 2023. (a) Biomass concentration (*C*_*x*_, circles), (b) average absorption cross section (*a*_*x*_, diamonds), (c) quantum yield (QY, squares), and (d) supernatant optical density at 400 nm (OD400, triangles). Dashed lines indicate the duration of the batches. Values are expressed as average ± standard deviation.

The oxygen-balanced mixotrophic pilot experiment, conducted over a span of 32 days in June and July of 2023, comprised three batches. Throughout these batches, various parameters were monitored through sampling and offline analysis to determine the growth and the physiological state of the culture. The parameters included biomass concentration (*C*_*x*_, g·L^–1^), absorption cross section (*a*_*x*_, m^2^·kg^–1^), photosystem II quantum yield (QY, *F*_v_/*F*_m_), and coproporphyrin III (COPROIII) concentration, a marker for anoxia, by measuring optical density at 400 nm. These analyses are presented in Figure 2.

The reactor was inoculated with a *C*_*x*_ of 0.5 g·L^–1^ (Figure 2A). Then, the biomass underwent a transition from autotrophy to a fixed glucose supply rate and then to oxygen-balanced mixotrophy (OBM) that lasted slightly more than a week. On day 9, automatic glucose feeding was initiated and the air input was switched off. *C*_*x*_ reached 4.3 g·L^–1^ within 2 days and stagnated around that level for 3 days more, after which we decided to dilute the reactor and start a new batch. In batches 2 and 3, the maximum *C*_*x*_ achieved were 7 and 6 g·L^–1^, respectively. Notably, no distinct stationary phase was observed in these batches, indicating that the cells were growing linearly for the whole time. These observations underscore two points: growth halt in the initial batch was not due to reaching a stationary phase caused by the light gradient but likely stemmed from another factor. The potential reasons for this are discussed in the following sections. Second, the subsequent dilutions in batches 2 and 3 did not induce photoinhibition, as evidenced by the resumption of linear growth post-dilution. This observation is also supported by the measurements of QY depicted in Figure 2C. No steep decrease was observed after dilution, and for most of the experiment, QY remained above 0.40. Additionally, the maximum values recorded at the end of batches 2 and 3, in the range of 0.60, are among the highest observed for this strain.^8,11,12^

Pigment content in the cells was indirectly monitored by measuring *a*_*x*_ (Figure 2B). Following inoculation, *a*_*x*_ increased from the initial values oscillating around 130–180 m^2^·kg^–1^, reflecting a response to a decrease in light penetration as cells grew. After both dilutions, *a*_*x*_ decreased to 140 m^2^·kg^–1^, corresponding to a sudden increase in light availability. Similar to the first batch, biomass growth in batches 2 and 3 resulted in an increase of pigment levels. In batch 2, *a*_*x*_ stabilized again slightly above 180 m^2^·kg^–1^, while in batch 3, it reached 165 m^2^·kg^–1^. These values are in line with previous lab-scale results for OBM cultivation in chemostat, where *a*_*x*_ stabilized at a value of 180 m^2^·kg^–1^.^12^ Because *C*_*x*_ was still increasing linearly, the stabilization of pigment content suggests that cells might have reached a threshold of light penetration beyond which pigments could not increase further.^14^ In the same chemostat study, the authors observed a higher value of *a*_*x*_ but only in autotrophic growth. It is plausible that the *a*_*x*_ measured in this study still constitutes the limit but only in glucose-adapted cells. Alternatively, a stable pigment level could result exclusively from long-term acclimation to natural light conditions.^15^ The combined result of reactor geometry and light intensity in the TPBR ultimately resulted in acclimation *a*_*x*_ levels that are similar to the observed levels in the lab-scale setup of these studies.

**Figure 3:**
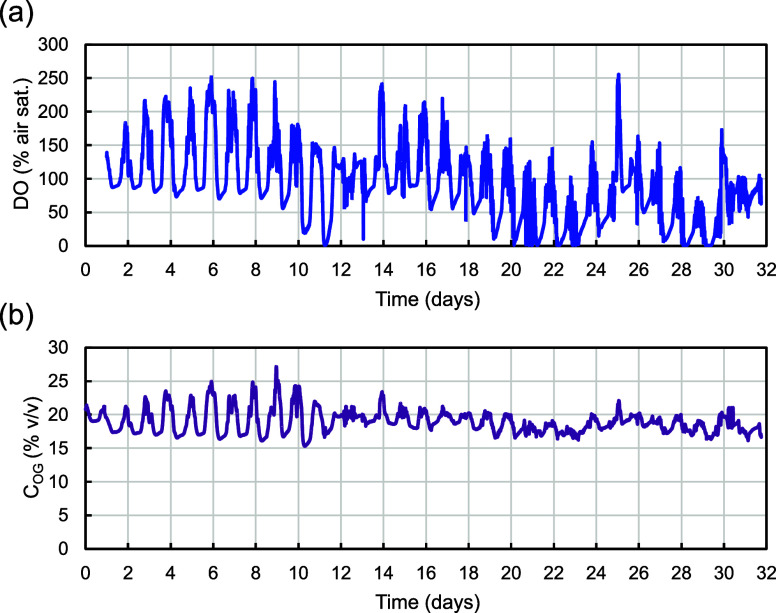
Online cultivation parameters during pilot-scale mixotrophic cultivation of *G. sulphuraria* in the summer of 2023. (a) Dissolved oxygen concentration and (b) oxygen concentration in the gas over time.

Finally, the concentration of COPROIII was followed by measuring the absorbance at 400 nm (Figure 2D). COPROIII is the oxidized form of coproporphyrinogen III, an intermediate in the synthesis pathways of chlorophyll and other pigments that is accumulated when cells undergo anoxic periods.^6^ Hence, it can be used as a marker of the O_2_ limitation. In all three batches, the initial concentration was very low, below 0.05. Throughout the experiment, levels of COPROIII remained largely stable, with the exception of instances where a steep rise was observed: from day 10 to 12, 20 to 24, and 28 to 30. Notably, these days coincide with periods of high biomass growth (Figure 2A) when also dissolved oxygen (DO) was depleted during the evening or at night (Figure 3A). This indicates an elevated night respiratory demand following periods of substantial growth, a phenomenon that has been observed before.^16^ Importantly, the maximum COPROIII levels did not exceed 0.2 in any batch. These observations suggest that the anoxic periods encountered by the cells did not exceed the threshold for causing damage.^6,17,18^

### 3.2 Controller Design and Performance

In the experiment, automatic control of glucose feeding was achieved via a PI controller based on the concentration of oxygen in the gas phase (*C*_OG_). In contrast to the development of OBM at lab scale, in this study, we selected the gas phase, and thus *C*_OG_, to control glucose supply instead of DO. *C*_OG_ is less prone to noise amplification than DO, albeit with some sluggishness attributed to the delay caused by mass transfer and mixing. The signal of DO might experience abrupter changes due to the biological activity occurring in the liquid phase. In addition, we decided to omit the derivative term (*D*) from the controller and utilize it solely as a PI. This choice was influenced by the errors derived from the amplification of the noise from the *C*_OG_ signal caused by the *D* term. A preliminary trial (data not shown) indicated that the PI controller yielded better results in managing these noise-induced errors.

During the first 2 days of OBM in the first batch (days 9–11) the control system worked successfully. However, from that point, until the dilution of the batch growth stopped despite light being still available. This trend can also be observed in the *C*_OG_ (Figure 3B). During this time, the controller was largely inactive and provided minimal glucose to the culture. This event coincided with a manual increase in night aeration on day 11 from 10 to 15 L·min^–1^. This increase was triggered by DO reaching 0 for the first time on that same day. The manual adjustment was aimed at preventing harmful anoxic effects in the biomass in the following nights. Eventually, we observed that supplying intermittently CO_2_ led to O_2_ production, and consequently, the controller would resume glucose feeding. This correlation pointed to the CO_2_ limitation as the probable cause of the growth halt.

Presumably, increasing night airflow to 15 L·min^–1^ led to excessive CO_2_ removal during the night, preventing the initiation of photosynthetic activity at the beginning of the next day. As a response, we optimized the initial glucose pulse at the beginning of the day by progressively increasing it each day until we achieved an effective trigger to counteract CO_2_ limitation. Starting at 2 mL·min^–1^ for 10 min per pump, we increased it to a final value of 12 mL·min^–1^ for 1 h per pump on day 18, maintaining this setting for the rest of the experiment. Also, on day 18, night aeration was reduced from 15 to 5 L·min^–1^ unless DO went below 10% air saturation, when the aeration rate was increased to the initial 10 L·min^–1^. In addition to the night aeration rate, the initial glucose pulse was also changed, because the initial trigger proved to be insufficient when the light input during sunrise was too low. In this manner, we allowed anoxic periods in exchange for avoiding CO_2_ limitation. Furthermore, on the same day, the *K*_P_ was increased from the initial value of 80 to 200 to ensure a robust start of the controller at sunrise and during the possible restarts during the day in the case of low light conditions. Additionally, the τ_I_ was decreased from the initial 2000 to 1500 s to promote a stronger controller response that prevented standstill periods. Nevertheless, this adjustment provoked oscillations in the glucose pumping rate that went from 0 to 100% without stabilizing. Because of this behavior, *K*_P_ was gradually reduced until a more stable response was obtained, settling at a value of 160 on day 23.

In conclusion, the preliminary controller settings served as a valuable starting point that reduced the time and efforts of completely manual optimization.^7^ Nevertheless, in the scenario in vivo the controller still required adjustments due to unforeseen factors that were not considered in previous studies, such as CO_2_ limitation because of excessive nighttime aeration. The initial settings of the controller worked moderately well but resulted in insufficient action. Interestingly, throughout the entire experiment, offline sampling showed that glucose concentration remained always below 50 mg·L^–1^, the detection limit of our equipment. Given the limitations of this method, we can still conclude that there were no indications of glucose accumulation. The results highlight the importance of simulating comprehensive scenarios beyond intuitive predictions or expectations. Simultaneously, a compromise between laborious computational design, time-consuming testing, and practical solutions that facilitate rapid controller design must be found.

### 3.3 Biomass Volumetric Productivity and Yield on Substrate at Pilot Scale

The primary characteristics of OBM include enhancing autotrophic productivity by up to 2-fold under conditions of comparable light input, coupled with achieving up to 90% substrate utilization efficiency.^1^ However, incrementing the cultivation volume might lead to suboptimal performance due to the scale-up effect.^19^ Furthermore, mixotrophy adds an additional layer of complexity to the scale-up associated with managing dynamic natural light conditions.

We analyzed the biomass productivity (*r*_*x*_, g·L^–1^·day^–1^) and the biomass yield on substrate (*Y*_*x*/s_, C-mol_*x*_·C-mol_s_^–1^) considering exclusively the OBM periods during the pilot experiment (Table 1). The average *r*_*x*_ was 0.81 g·L^–1^·day^–1^, representing a nearly 50% reduction compared to the results obtained previously in chemostat and in repeated batch with OBM at lab scale, 1.66 g·L^–1^·day^–1^.^8,12^ It is worth noting that in those studies, the cultures were illuminated for 24 h per day, while in the current study, the cells experienced natural day/night cycles. If the *r*_*x*_ is normalized by 16 h of daylight, the result would be 1.22 g·L^–1^·day^–1^, which is only around 25% less than the reference.

**Table 1:** Overview of Average Process Parameters during Pilot-Scale Mixotrophic Cultivation of *G. sulphuraria* in the Summer of 2023^a^

	unit	value
temperature	°C	36.8 ± 1.2
pH		1.7 ± 0.1
DO	% air saturation	90.8 ± 53.1
*C*_OG_	% v/v	18.9 ± 1.7
*r*_*x*_	g·L^–1^·day^–1^	0.81 ± 0.15^b^
*Y*_*x*/s_	C-mol_*x*_·C-mol_s_^–1^	0.68 ± 0.03^b^

a Values expressed as average ± standard deviation.

b The calculation only considered periods under oxygen-balanced mixotrophy.

**Figure 4:**
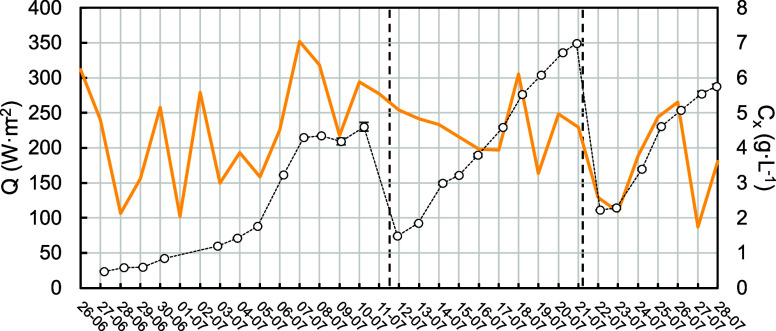
Average daily incoming shortwave radiation (*Q*, solid line) and biomass concentration (*C*_*x*_, circles) during pilot-scale mixotrophic cultivation of *G. sulphuraria* in the summer of 2023. Dashed lines indicate the duration of the batches. Values are expressed as averages ± standard deviation where applicable.

On the most productive periods during the experiment, encompassing days 8–10, 17, 20–21, and 27–29, *r*_*x*_ exceeded 1 g·L^–1^·day^–1^, reaching a *r*_*x*_ as high as 1.25 g·L^–1^·day^–1^. If these values were normalized similarly, they would match or even improve lab results by 13% in the best case. The periods of high *r*_*x*_ correlate well with an increase in radiation levels (Figure 4), except for day 17, corresponding to the 13th of July. Furthermore, the average *r*_*x*_ obtained in this study also compares favorably with another mixotrophic pilot system previously tested with *G. sulphuraria* ACUF 064, an annular column of 17 L.^11^ In that reactor, continuous artificial illumination was maintained for 24 h per day, and glucose was provided at a fixed rate, resulting in a *r*_*x*_ of 0.55 g·L^–1^·day^–1^. We also conducted an initial pilot-scale cultivation experiment under OBM during the summer of 2022. Although technical difficulties prevented the experiment from running smoothly, we achieved an *r*_*x*_ of 0.71 ± 0.43 g·L^–1^·day^–1^ during the OBM phase. These values are consistent with the results obtained in the 2023 experiment described in detail in this manuscript, further supporting our findings. Additional information about this trial is available in the Supporting Information.

**Figure 5:**
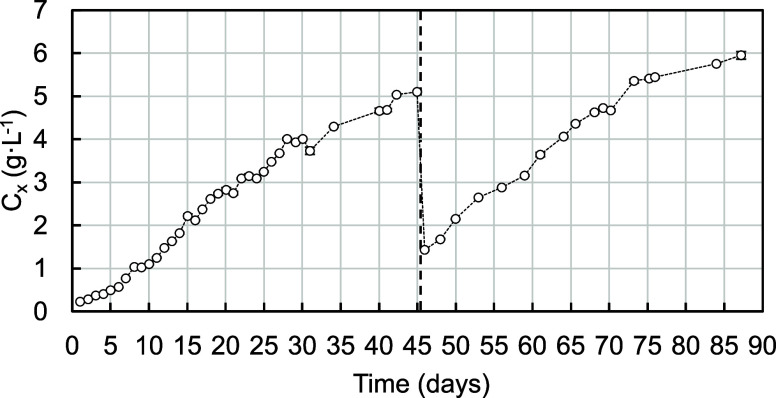
Biomass concentration during pilot-scale autotrophic cultivation of *G. sulphuraria* in the summer of 2021. Dashed lines indicate the duration of the batches. Values expressed as average ± standard deviation.

To better contextualize these findings, the *r*_*x*_ obtained with OBM can be compared with the *r*_*x*_ from an autotrophic experiment with the same strain and TPBR in the summer of 2021 (Figure 5). This experiment was carried out for nearly 3 months in 2 different batches. During the linear growth phases of both batches, the average productivity was 0.12 ± 0.13 g·L^–1^·day^–1^, reaching peaks within the 0.30–0.40 g·L^–1^·day^–1^ range. It is important to emphasize that in this experiment, temperature control was less stringent due to the utilization of a less powerful heat exchanger. The recorded average temperature was 30.0 ± 3.9 °C, which deviates further from the optimum of 37 °C and incurs a performance cost.^20^ Apart from that difference, natural light conditions are considerably variable between summer seasons. Despite these challenges, the results clearly demonstrate that OBM in a pilot-scale TPBR enhanced autotrophic rates. This improvement was obtained without the need for gas exchange during the day but at the expense of the organic carbon source. It is also apparent that the *r*_*x*_ under both OBM and autotrophic cultivation is significantly influenced by light availability. However, in the case of OBM, heterotrophic metabolism can proceed in the dark if the oxygen in aqueous O_2_ is available. In that regard, *r*_*x*_ observed in the mixotrophic experiment showed less variability.

Concerning *Y*_*x*/s_, the calculation result over all OBM phases in the experiment was 0.68 ± 0.03 C-mol_*x*_·C-mol_s_^–1^. This marks a 25% reduction over the maximum theoretical OBM yield of 0.90, previously achieved at lab scale with *G. sulphuraria*.^12^ This reduction aligns with the *Y*_*x*/s_ obtained in our first mixotrophic pilot test during the summer of 2022. In that experiment, we achieved a yield of 0.74 ± 0.01 C-mol_*x*_·C-mol_s_^–1^, although over a short period (see the Supporting Information). These results position within the range of the least favorable scenario tested in our previous scale-down study, which showed a 22% reduction.^6^ In that case, the low *Y*_*x*/s_ was attributed to the combined effect of cyclic oxygen depletion and glucose fluctuations, which triggered carbohydrate and protein excretion into the medium. Although DO levels were occasionally depleted in the current experiment, these periods were brief and did not show a recurring pattern (Figure 3A). Moreover, considering the results of COPROIII obtained in this experiment (Figure 2D), it appears that these periods of O_2_ limitation did not have a large influence in the biomass, in contrast to the scale-down scenario in our previous study. Notably, the aforementioned periodic glucose measurements throughout the experiment showed that glucose was completely consumed. These measurements rule out the possibility of having a decrease in the observed *Y*_*x*/s_ due to unconsumed glucose. Unfortunately, the scope of this study did not encompass the monitoring of organic carbon in the medium.

The decrease in *Y*_*x*/s_ could potentially be attributed, at least partially, to additional factors specific to this scenario. The results obtained in the laboratory in our previous study were acquired under continuous illumination, whereas in the current scale-up experiment, cells encountered day/night cycles. Although the precise effect of day/night cycles on *Y*_*x*/s_ is unknown, it is reasonable to anticipate a reduction in glucose utilization efficiency due to biomass losses during nighttime.^21^ Another plausible explanation is that the peaks in light, CO_2_, O_2_, and glucose availability may not have been perfectly synchronized within the cells. This asynchrony may lead to cell-specific changes in metabolic adaptations, potentially redirecting carbon toward energy production and resulting in the overall loss of CO_2_ that could not be efficiently recycled.^22^ Thus, excess CO_2_ generated from increased energy production would have been released into the gas phase. Due to technical constraints in our setup, measuring the CO_2_ concentration in the off-gas was unattainable, and this hypothesis could not be tested. Therefore, the potential scenarios of organic carbon being excreted into the liquid phase or the additional carbon being utilized for energy generation both remain plausible. Lastly, we can dismiss the influence of contaminating microbes in glucose consumption. While filamentous fungi were occasionally detected in the culture, their presence was minimal and we observed no significant population increase.

Even so, the glucose supply strategy still contributed to CO_2_ recycling, with 70% of the carbon in glucose being directed toward biomass. This efficiency notably enhances the heterotrophic yield on substrate of *G. sulphuraria*, previously measured at 0.59 C-mol_*x*_·C-mol_s_^–1^.^8^ Moreover, this result approaches the theoretical maximum yield achievable in chemoorganoheterotrophic processes,^23^ seldom achieved at large scales, and marks a promising milestone for OBM. Gaining deeper insights into the complex dynamics of the process is essential to refine the control strategy and potentially improve the results on a large scale. This research illustrates the feasibility of achieving OBM under industrially relevant conditions, using a 280 m two-phase TPBR as an example. The evaluated tube length can be expanded without major changes to the conditions experienced by the cells through the arrangement of multiple tubular sections of identical lengths in parallel. Therefore, it can be concluded that the technology is prepared for industrial application.

## 4 Conclusions

The scale-up of oxygen-balanced mixotrophy (OBM) with *G. sulphuraria* ACUF 064 was successfully achieved in a pilot two-phase tubular photobioreactor (TPBR) of 1700 L with natural light supply. Glucose feeding was regulated by a proportional-integral controller that followed the concentration of oxygen in the continuously recirculated gas phase of the system. We discovered that minimizing aeration during the night is crucial to preventing excessive carbon dioxide (CO_2_) loss. Additionally, introducing a pulse of glucose at the beginning of the day is essential to effectively initiate the mixotrophic cycle. The average mixotrophic biomass productivity was 0.81 g·L^–1^·day^–1^, demonstrating a considerable improvement over the autotrophic productivity in the same TPBR, which was an average of 0.12 g·L^–1^·day^–1^. Mixotrophic productivity exhibited a considerable correlation with the availability of natural light. The biomass yield on substrate over the OBM periods during the experiment was 0.68 C-mol_*x*_·C-mol_s_^–1^, reflecting a 25% reduction from the optimal lab-scale results but still high in comparison to heterotrophic cultivation. This decline might be attributed to carbon redirection toward energy production and extracellular biomolecules triggered by fluctuations in oxygen, CO_2_, glucose, and light availability. Despite observing inefficiencies at pilot scale, these findings demonstrate that carbon is recycled to a significant degree. The two-phase tubular system proved to be well suited for OBM, demonstrating the readiness of the technology for application. This study paves the way for further optimization and implementation of the process at a larger scale.

## Supporting Information Available

Mixotrophic pilot-scale cultivation of *Galdieria sulphuraria* in the summer of 2022 (PDF)
