# Supplementary material for: Pilot-Scale Oxygen-Balanced Mixotrophic Cultivation of Galdieria sulphuraria
Source: ACS Sustain Chem Eng. 2025 Jan 31;13(5):2132–40. doi: 10.1021/acssuschemeng.4c09186 (PMC11816015; doi:10.1021/acssuschemeng.4c09186)
Supplement: Supplementary file 1 — sc4c09186_si_001.pdf [file sc4c09186_si_001.pdf]

Supporting Information

Pilot-scale oxygen-balanced mixotrophic cultivation of *Galdieria sulphuraria*

Pedro Moñino Fernández[1]*, Marina López Morales*[1]*, Aniek de Winter*[1]*, Fred van den End*[1]*, Marcel Janssen*[1*]*, Maria Barbosa*[1]

[1]Bioprocess Engineering, AlgaePARC, Wageningen University and Research, P.O. Box 16, 6700 AA Wageningen, The Netherlands

*Corresponding author: marcel.janssen@wur.nl

4 pages, 1 figure

Supporting Information 1: Mixotrophic pilot-scale cultivation of *Galdieria sulphuraria* in the summer of 2022

Galdieria sulphuraria ACUF 064 was cultivated under oxygen-balanced mixotrophy in a tubular photobioreactor (*TPBR*) GemTube MK-1 1500s (Lgem, the Netherlands) located at AlgaePARC (Bennekom, the Netherlands). The experiment was conducted over 35 days during late spring and summer of 2022, from the 17th of June to the 22nd of July. The two-phase reactor was operated with a starting working liquid volume of approximately 1350 L and using exclusively natural light. The medium had an initial pH of 1.8, and no active pH control was applied throughout the experiment. For the first 10 days after inoculation, the *TPBR* was operated autotrophically to allow the cells to adapt to the new flow and light conditions. Following this period, the transition to oxygen-balanced mixotrophy (*OBM*) began, with a 200 $g \cdot L^{-1}$ glucose solution supplied at a constant rate of 1.7 $mL \cdot min^{-1}$ to acclimate cells to glucose metabolism. After 4 days in this regime, the glucose supply control based on oxygen concentration in the gas phase started with the same glucose solution. The supply was controlled by a *PI* controller. During nighttime glucose supply was stopped. *OBM* was maintained until the end of the batch and in the second and final batch, for a total of 13 and 8 days, respectively. The culture was regularly monitored, including dry weight measurements (Figure S1), and contamination was checked weekly. *DO* and C_{OG} were monitored by online measurements.

The temperature was controlled between 26 and 50 ºC using a 3 kW heat exchanger and partial greenhouse temperature regulation, resulting in an average temperature of 32.6 ºC with a standard deviation of 5.0. The heat exchanger also provided cooling when required. Gas and liquid recirculation were initially facilitated by a single gas compressor and a liquid recirculation pump, respectively. This setup achieved a gas recirculation flow rate of 20 $L \cdot min^{-1}$ and a liquid velocity of around 0.4 $m \cdot s^{-1}$. In addition to gas recirculation,

an airflow enriched with 10% v/v CO_2 was supplied continuously at 10 L·min^{-1}, along with an equivalent gas bleed flow rate, both during day and night. Due to the limited capacity of the heat exchanger, the *TPBR* occasionally exceeded 45 ºC, which temporarily halted the gas compressor and caused periods of up to 45 minutes without mixing until the system could restart. During these intervals, glucose supply was also paused. Additionally, the liquid pump failed on day 11 due to overheating, after which the *TPBR* was operated solely with the gas compressor. Consequently, the liquid velocity dropped to a range of 0.1 – 0.2 m·s^{-1} for the remainder of the experiment, resulting in a decoupling of the liquid and gas residence times. This adjustment also altered the gas holdup, resulting in a working volume of approximately 1150 L for the rest of the operation.

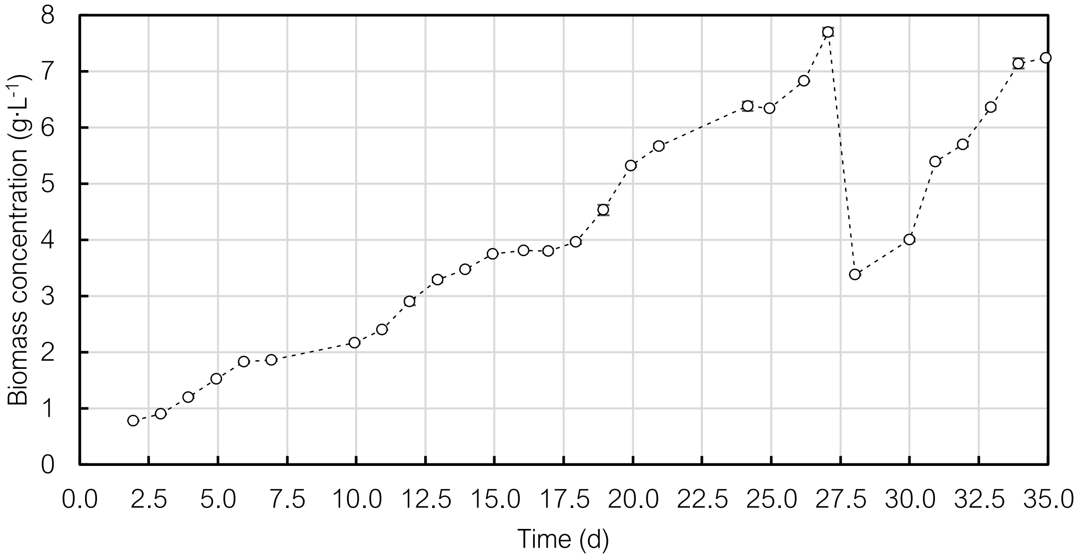

Figure SI1. Biomass dry weight concentration during pilot-scale mixotrophic cultivation of *Galdieria sulphuraria* in the summer of 2022. Values expressed as averages ± standard deviation.

Due to frequent technical interruptions during batch 1 and the subsequent shift to operation exclusively with gas compressor, we do not consider it representative for calculating volumetric productivity (r_x, g·L^{-1}·day^{-1}) and biomass yield on substrate ($Y_{x/s}$,

$C\text{-mol}_x \cdot C\text{-mol}_s^{-1}$). In addition, the initial configuration of the glucose supply *PI* controller was adjusted to enhance the responsiveness of the system, with the final parameters being established at the start of batch 2. Instead, we based these calculations exclusively on batch 2, which was conducted entirely under *OBM* conditions. r_x during this period of 7 days was an average of 0.71 ± 0.43 $g \cdot L^{-1} \cdot day^{-1}$ and $Y_{x/s}$ was 0.74 ± 0.01 $C\text{-mol}_x \cdot C\text{-mol}_s^{-1}$.
